# Biotransformation of gallate esters by a pH-stable tannase of mangrove-derived yeast *Debaryomyces hansenii*

**DOI:** 10.3389/fmolb.2023.1211621

**Published:** 2023-06-08

**Authors:** Lei Song, Xiao-Chen Wang, Zhen-Quan Feng, Yan-Feng Guo, Guo-Qing Meng, Hai-Ying Wang

**Affiliations:** ^1^ College of Agriculture and Bioengineering, Heze University, Heze, China; ^2^ Department of Clinical Laboratory, University of Health and Rehabilitation Sciences (Qingdao Municipal Hospital), Qingdao, Shandong, China; ^3^ College of Ecological and Environmental Protection, Linyi Vocational University of Science and Technology, Linyi, China; ^4^ Key Laboratory of Sustainable Development of Polar Fishery, Ministry of Agriculture and Rural Affairs, Yellow Sea Fisheries Research Institute, Chinese Academy of Fishery Sciences, Qingdao, China

**Keywords:** tannase, pH stability, tannin biodegradation, gallate esters, gallic acid

## Abstract

**Introduction:** Tannase is a crucial enzyme that finds wide applications in the pharmaceutical industry, feed processing, and beverage manufacturing. Although extensive studies have been conducted on tannases from fungi and bacteria, reports on tannases exhibiting favorable pH stability are relatively limited.

Methods: In this study, a tannin-degrading strain *Debaryomyces hansenii* was screened to induce tannase production, and the corresponding tannase coding gene TANF was successfully cloned and expressed in *Yarrowia lipolytica*. SDS-PAGE analysis revealed that the purified TanF tannase had a molecular weight of approximately 70 kDa.

**Results and Discussion:** The enzyme demonstrated optimal activity at 40°C and retained over 80% of its activity in the range of 35°C–60°C. Of particular interest, TanF exhibited remarkable enzyme activity at pH 5.0 and retained more than 70% of its relative activity across a wide pH range of 3.0–8.0. Furthermore, TanF exhibited broad substrate specificity for gallate esters. The final gallic acid production by TanF from tannic acid achieved 18.32 g/L. Therefore, the excellent properties TanF has been demonstrated to be an efficient tool for the preparation of gallic acid.

## Introduction

Tannins are a type of polyphenolic compounds that are abundant in higher plants, with a molecular weight ranging from 500 Da to 28,000 Da. After cellulose, hemicellulose, and lignin, tannins are the fourth most abundant substance in plants. The presence of phenolic rings and phenol hydroxyl groups in tannins enables them to capture metal ions and form strong interactions with macromolecules such as proteins, cellulose, starch, and other polysaccharides. The resulting complexes can lead to noticeable insolubility and precipitation effects (Ragan, 1986; Schofield et al., 2001; Abebe et al., 2007; Jeong et al., 2013). Chemically, tannins can be classified into three major categories: phlorotannins, condensed tannins, and hydrolyzable tannins (Ragan, 1986). Condensed tannins primarily accumulate in the endothelial layer of rapeseed between the aleurone layer and the outer integument, which contributes to the darker color of the seed coat. Tannins have an unpleasant taste, astringent properties, and are indigestible, and these properties enable them to play a role in the defense against invading pathogenic microorganisms and herbivores by forming stable complexes with biological macromolecules (Buzzini et al., 2008).

Protein digestibility is a critical factor in determining the bioavailability of protein in rapeseed meals (Fuller and Tomé, 2005). Recently, various methods have been employed to degrade tannins and improve protein digestibility *in vitro*. Heat processing is a practical technique that can enhance protein digestibility, but it often results in nutrient loss (Petitot et al., 2009). Lye soaking solution (Salem et al., 2005; Canbolat et al., 2007) and high-pressure treatment (Xue et al., 2020) have been utilized for tannin degradation and protein digestibility improvement, but their widespread use is limited by high equipment requirements and costs.

Compared to other methods, enzymatic hydrolysis is a mild and effective degradation method that can efficiently degrade anti-nutritional factors in oilseed meals, enabling the protein to be fully utilized (Nair and Duvnjak, 1990; Hao et al., 2020; Wang et al., 2022). However, studies on the microbial degradation of tannins in rapeseed meals are scarce. Some microorganisms are known to be resistant to tannins and produce tannin-degrading enzymes. Tannase (EC 3.1.1.20), also known as tannin acyl hydrolase, directly cleaves the ester bonds in all types of tannins, including gallotannins, ellagitannins, and gallic acid esters. The product of tannase-based degradation is gallic acid and ellagic acid (Ebune et al., 1995; Tripathi and Mishra, 2007; Lomascolo et al., 2012; Mejicanos et al., 2016). Tannase-based degradation of tannins has received significant attention. Li et al. reported that tannase generated through *Trametes versicolor* was utilized to treat rapeseed meals, and the results indicated that the degradation rate of tannins exceeded 80% within 30 min (Lorusso et al., 1996). Tannase has also been used to prevent chill haze in beer production.

The biotransformation of gallic acid from gallotannins through tannase catalysis has gained increasing importance as a significant application of tannase. This process has been extensively studied by various researchers (Bell, 1984; Lacassagne et al., 1988; Al-Mamary et al., 2001; Şengil and Özacar, 2009; El-Beltagi and Mohamed, 2010; Frazier et al., 2010). Gallic acid, a product of tannin degradation, has been found to possess a range of biological activities, such as anticarcinogenic, antiallergic, antibacterial, anti-inflammatory, and antioxidant properties (Lacassagne et al., 1988; Frazier et al., 2010). Its medicinal properties make it a potential candidate for drug material and food additives. Gallic acid has also been used as a noted medical intermediate in the pharmaceutical industry for synthesizing trimethoprim, a commercial antibacterial drug, and specific gallate esters, which serve as green food preservatives (Lacassagne et al., 1988; Al-Mamary et al., 2001; Abebe et al., 2007; Şengil and Özacar, 2009).

Although the chemical method of producing gallic acid through acid hydrolysis has been widely used, but it has some limitations in terms of its purity and yield. In contrast, the enzymatic method, which utilizes tannase, has demonstrated higher efficiency, purity, and yield with less environmental pollution. However, there is still a dearth of information on the bioconversion of tannic acid to gallic acid *via* tannase (Lacassagne et al., 1988; Al-Mamary et al., 2001; Abebe et al., 2007; Ozturk et al., 2016). To ensure that tannase can effectively catalyze the hydrolysis of its substrate, tannic acid, it is necessary to maintain a low pH during the reaction. Few characterized tannases thus far exhibit high catalytic activities within a narrow pH range, which is insufficient for gallic acid production (Kanpiengjai et al., 2020). Therefore, tannases working over the broad pH range is demanded in application.

Mangroves, being a typical transitional ecosystem, possess a rich diversity of species, including microorganisms. These microorganisms, due to their proximity to both terrestrial and marine habitats, have been found to secrete degrading enzymes that can utilize plant materials as nutrient substances (Zhang et al., 2019; Wang et al., 2022). In this study, a novel tannase abbreviated as TanF was successfully obtained from *Debaryomyces hansenii* and expressed effectively in yeast host. This enzyme demonstrated superb pH stability and a unique ability to degrade tannin, making it a potent tool for tannin biodegradation and gallic acid production.

## Materials and methods

### Strain screening and gene cloning

Leaf and soil samples from mangrove environments (Leqing, Zhejiang Province) were uniformly scattered and cultured in YPT medium, containing 10 g/L peptone, 5 g/L glucose, 15 g/L tannic acid, and 0.002 g/L bromophenol blue, pH = 3.0 (30). Then the culture solution was diluted and spread on YPT plates. The isolated strains growing on YPT plates were tannin-tolerant and can produce tannase. To complete the strain identification, universal primers were used to amplify the 26S rDNA of the strain, which was subsequently sequenced and aligned with BLAST. In order to identify the genetic code of the tannase from strain *Debaryomyces hansenii* Y71. The genomic DNA was sequenced and annotated to obtain the annotation information about the tannin-degrading enzymes, the gene sequence was compared with the Carbohydrate-Active enzymes (CAZy) database (Pan et al., 2020).

### Activity determination

In this article, the activity of tannase was measured by determining the production of gallic acid generated by degrading propyl gallate. Then gallic acid can react chemically with rhodamine, generating purple chromogen with maximum absorption peak at 520 nm (Sharma et al., 2000). Firstly, the propyl gallate substrate was prepared in phosphate buffer (20 mM) with a pH of 5.5 and a concentration of 0.8% (w/v). The substrate solution (700 µL) was pre-heated at 50 °C, and then 50 µL of enzyme solution was added to maintain for 10 min. The reaction mixture was boiled for 10 min to terminate the reaction, the reaction mixture using boiled enzyme solution was set as the control. After the 10 min-reaction, 150 μL rhodamine methanol solution with a concentration of 0.667% (w/v) was added into the mixture and incubated at 30 °C for 5 min. Then, accompanied by the addition of 100 μL 0.5 M KOH solution, the purple complex appeared. The final mixture was kept at 30 °C for extra 10 min. Then the absorbance at 520 nm was determined. The amount of generated gallic acid was calculated according to the standard curve, which link up the OD_520_ value and gallic acid concentration. The tannase activity unit (U) was defined by the specific amount of the enzyme, which can catalyze the reaction with the increasement of 0.1 μmol in 1 min.

### Bioinformatics analysis of TanF

Annotation and sequencing were performed on the genomic DNA (Novogene, China) to acquire the coding sequence of current tannase. The result revealed the existence of a puta-tive tannase gene with an open reading frame (ORF) of 1767 bp. Reduced repetition (http://www.dtu.dk/services/SignalP/), and Conserved Domain Database was used to execute domain analysis (https://www.ncbi.nlm.nih.gov/cdd). Isoelectric point (pI) and theoretical molecular weight (Mw) were acquired *via* the website (https://web.expasy.org/compute_pi/). Phylogenetic tree was produced applying neighbor connection means in MEGA version 7.0 according to the reported tannins.

### Secretory expression of TanF

When the codon has been optimized, the gene synthesis embodying signal peptide was completed by Synbio Technologies, which was further transformed into the uracil defect type strain URA-. After 36-h culturing in GPPB media at 35°C, the positive transformant signal of TanF tannase activity were detected (Zhang et al., 2018). Additional, after the strain with the optimal extracellular activity was fermented in a flask for 96 h, the supernatant was then subjected to gel filtration chromatography column (GE Healthcare, United States) after adjusting the pH to 5.0. In the following of eluting with imidazole solution, sodium dodecyl sul-fate-polyacrylamide gel electrophoresis (SDS-PAGE) was applied to determine the purity and Mw (Molecular weight) of TanF.

### Temperature and pH properties of TanF

In order to obtain the optimal reaction temperature of present tannase, the catalytic hydrolysis reaction participated with TanF was completed in NaOH-glycine (10 mM) buffer within the range of 20°C–70°C. For the thermostability study of TanF, after cultured at 20°C–70°C for 10 h, the residue activity of tannase was confirmed in an environment of 40°C. Buffers (NaOH-glycine, pH 8.0–11.0; citric acid-Na_2_HPO_4_, pH 2.0–8.5) were applied to prepare tannic acid solutions to conduct as the substrates for confirming the optimum reaction pH of TanF. The pH stability was studied by means of measuring the residual enzyme activity after 12 h incubation at 40°C in different pH buffers. All measurements were repeated three times.

### The properties of TanF affected by various metal ions and chemicals

Prepare the tannic acid solutions containing metal ions/compounds at a concentra-tion of 1 mM, respectively. The tannase-catalyzed hydrolysis reaction was carried out at 50°C to appraise the effect of these substances on the activity of TanF. The reaction per-formed in the tannic acid solution not containing the above-mentioned substances served as the blank group. All measurements were repeated three times.

### Substrate specificity of TanF

To determine the substrate specificity of TanF, various gallate esters [including methyl gallate (MG), epicatechin gallate (ECG), propyl gallate (PG), catechin gallate (CG), epigallocatechin gallate (EGCG), and tannic acid (TA)] were used as substrates for the enzyme. The amount of gallic acid produced by TanF was determined by measuring the absorbance at 520 nm. Additionally, the content of gallic acid and esters was estimated using HPLC. HPLC conditions included the use of an Agilent SB-C18 column (10 μm, 3.0 × 250 mm), mobile phases A (water-0.5% acetic acid) and B (acetonitrile), and a flow rate of 0.8 mL/min. Gradient elution was employed (Ni et al., 2015).

### Gallic acid production from tannic acid catalyzed by TanF

The recombinant TanF liquid was concentrated 10-fold by ultrafiltration, to make the final liquid with an activity of 200 U/mL. Different TA solutions (10 g/L, 20 g/L, and 30 g/L) were prepared with a pH of 5.0, as the substrates for gallic acid production. 5 mL TanF liquid was added into 1,000 mL TA solution and incubated at 50°C for 12 h. Samples were taken every hour to detect gallic acid content.

## Results

### Bioinformatics analysis of the tannase TanF

**FIGURE 1:**
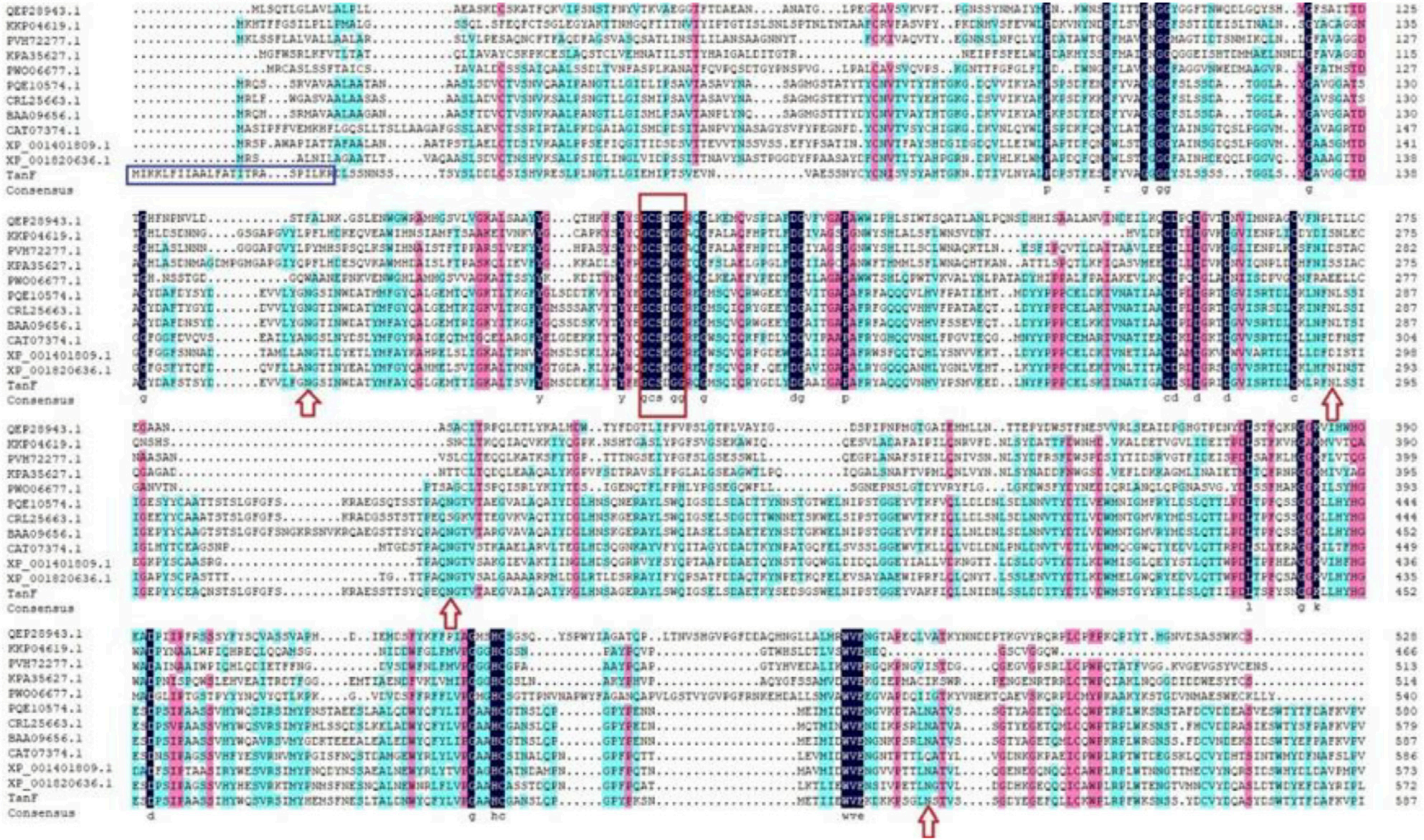
Multiple sequence alignment among TanF and other reported tannases. The signal peptide is boxed in blue, the Gly-X-Ser-X-Gly conserved domain is highlighted by a red box, and the predicted N-glycosylation sites are marked with red arrow.

**FIGURE 2:**
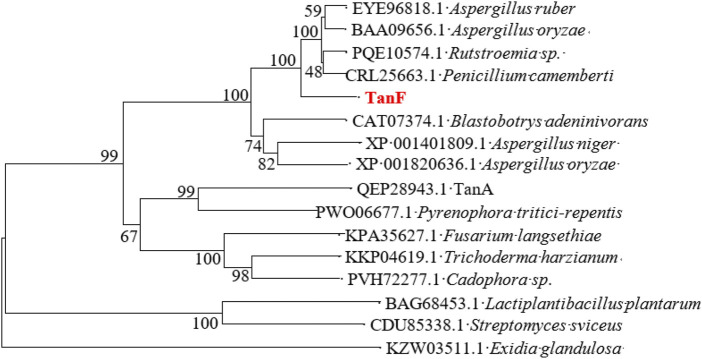
The phylogenetic tree constructed according to the sequences of TanF and other tannases. TanF researched in this study is in red.

After isolation and purification, 13 tannin-tolerant strains were obtained. Strain Y71 was found to with highest tannase activity of 14.3 U/mL. The strain was identified as *Debaryomyces hansenii.* The yeast strain Y71 can be helpful resource for degradation of tannins. To clone the gene encoding of present tannase, we complete its DNA sequencing genome, with the result displaying there is a putative gene encoding tannase, abbreviated as *TANF*. The ORF was made up of 1767 bp as well as encoded a protein consisting of 588 amino acids. Further biological analysis shows that the first 24 amino acids (as shown in the blue box of Figure 1) of TanF are projected to be signal peptides, which is according to the secretion traits. The theoretical PI and Mw of the signal peptide are forecasted to be 4.50 and 62.92 kDa, respectively. To further confirm the ascription of TanF, the phylogenetic tree is built based on the amino acid sequences of reported tannase (Figure 2). The 11 representative enzymes contain tannase from *Aureobasidium melanogenum* (Accession: QEP28943.1), tannase from *Cadophora* sp. (Accession: PVH72277.1), tannase from *Trichoderma harzianum* (Accession: KKP04619.1), tannase from *Fusarium langsethiae* (Accession: KPA35627.1), tannase from *Rutstroemia* sp. (Accession: PQE10574.1), tannase from *Pyrenophora tritici-repentis* (Accession:PWO06677.1), tannase from *Penicillium camemberti* (Accession: CRL25663.1), tannase from Aspergillus ruber *Aspergillus oryzae* (Accession: BAA09656.1), tannase from *Blastobotrys adeninivorans* (Accession: CAT07374.1), tannase from *Aspergillus niger* (Accession: XP_001401809.1), tannase from *A. oryzae* (Accession: XP_001820636.1). Therefore, these analysis results indicate that TanF possesses clear catalytic activity or can effectively bind the substrates of tannic acid.

### Recombinant expression of TanF in *Y. lipolytica*

**FIGURE 3:**
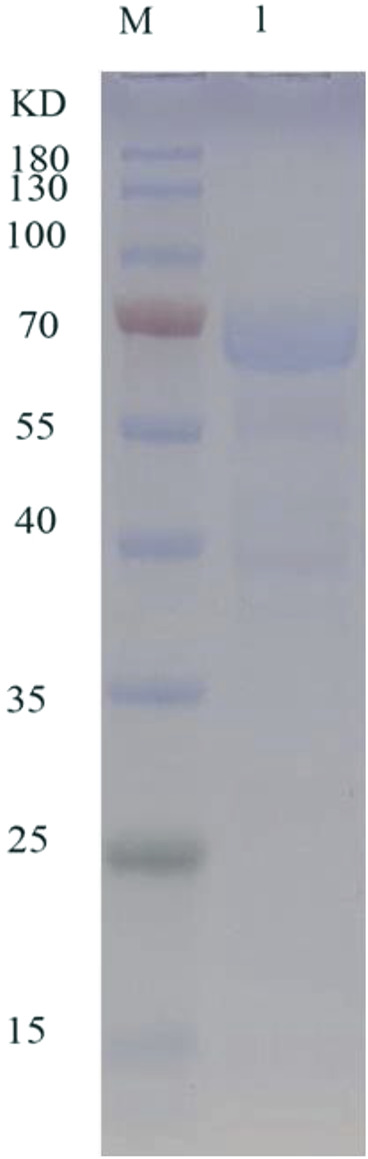
SDS-PAGE of purified TanF. Lane 1: purified TanF; lane M: prestained protein ladder.

In the detailed work of this paper, TanF has been successfully expressed in the common heterologous host, namely *Yarrowia lipolytica*, and achieved outstanding extracellular secretion (Madzak, 2015). After incubation in GPPB culture medium, the tannase activity is determined as 36.3 U/mL. Figure 3 reveals the result of SDS-PAGE analysis of TanF protein. This figure shows a clear single band, indicating that the Mw of TanF is about 65.0 kDa, slightly bigger than the theoretical value, which may be caused by glycosylation of proteins. For that, the NetNGlyc 1.0 server has been used to predict the four N-linked glycosylation sites of TanF (as shown by the red arrows in Figure 1). Additionally, several additional amino acids are introduced into the recombinant TanF by 6×His-tag.

### Temperature and pH properties of TanF

**FIGURE 4:**
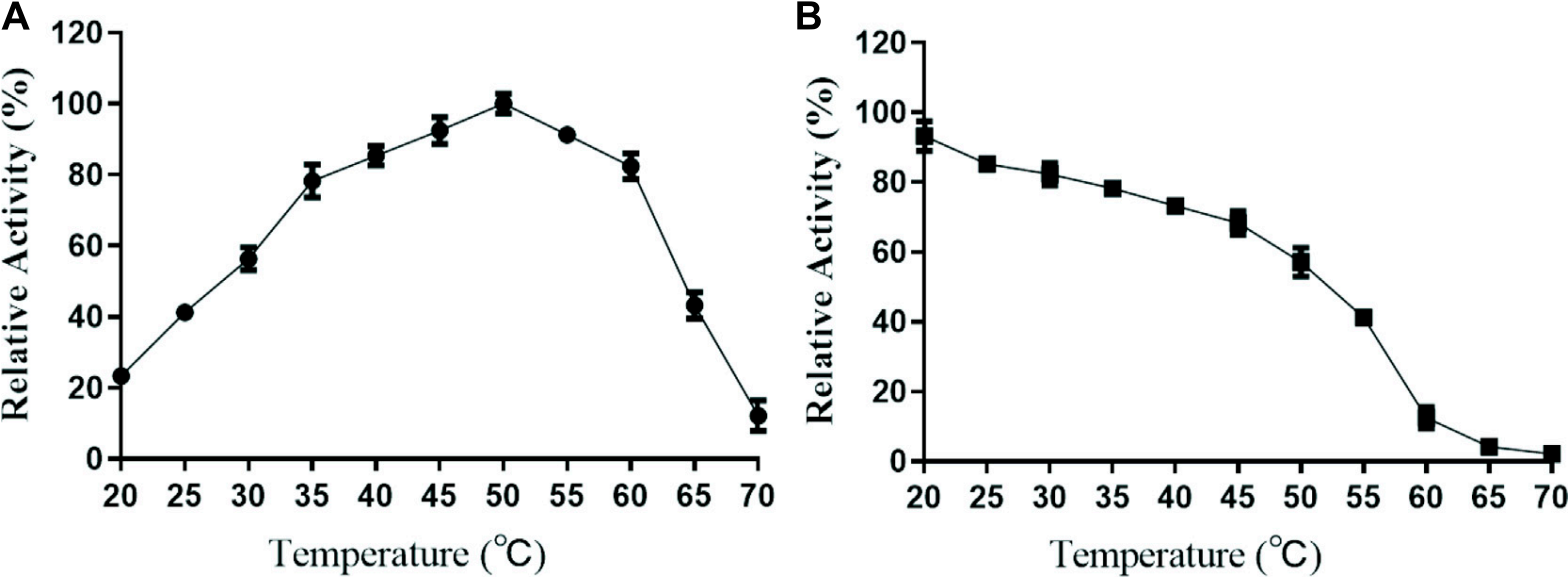
Effects of temperature on the activity **(A)** and stability **(B)** of TanF. **(A)** The optimal temperature of TanF was assessed in the range of 20°C–70°C, regarding the activity at optimum temperature as 100%. **(B)** The temperature stability of TanF was determined by measuring the residual activity after incubation under 20°C–70°C for 12 h; the initial activity was taken as 100%, *n* = 3.

In this study, the properties of purified TanF have been determined at different temperatures and pH conditions. As revealed in Figure 4, the TanF shows more than 80% of the highest catalytic activity at 35°C–60°C with the highest activity at 50°C, while drops sharply when the temperature exceeded 60°C. It should be noted that even at 60°C and 65°C, the present enzyme still possesses 80.9% and 42.4% of the relative catalytic activity, showing a certain heat resistance.

**FIGURE 5:**
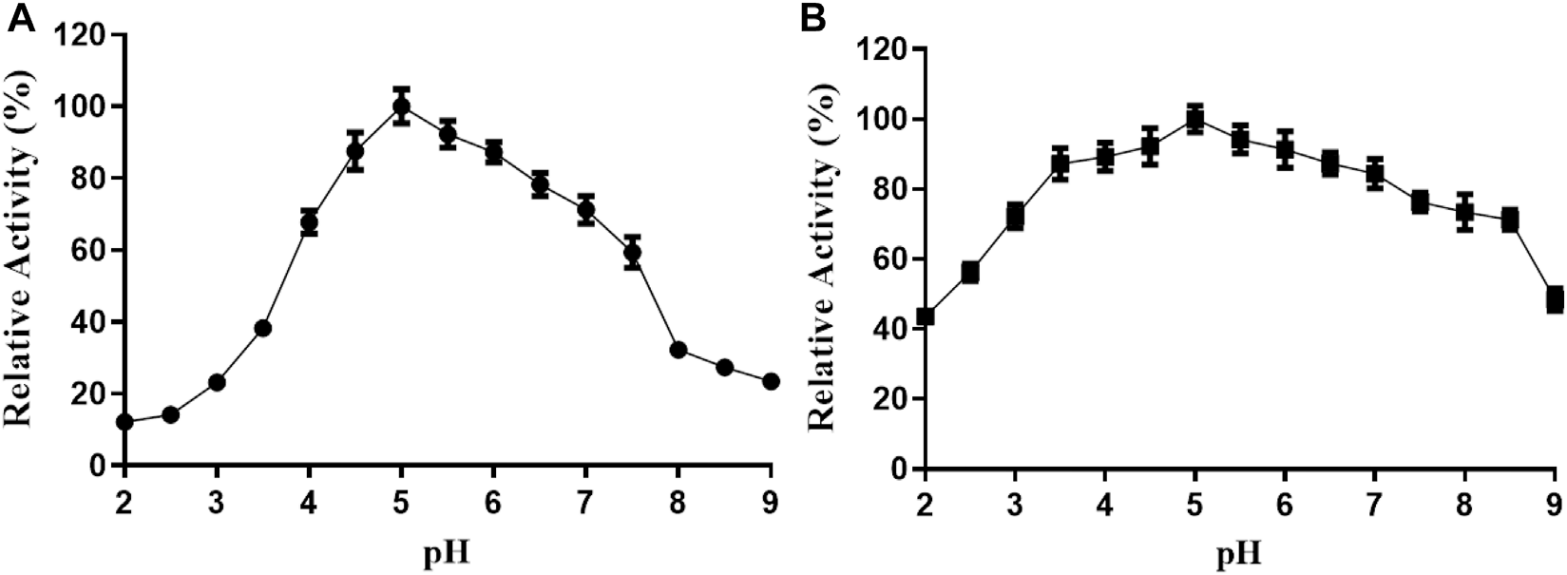
**(A)** Effect of pH on the TanF activity was determined by performing an activity assay in buffers (NaOH-glycine, pH 8.0–11.0; citric acid-Na_2_HPO_4_, pH 2.0–7.0). **(B)** Effect of pH on the TanF stability was determined by incubation at different pH values. Data are expressed as mean ± standard deviation, *n* = 3.

The activity of TanF tannase is further investigated under different pH conditions. As shown in Figure 5A, TanF displays the highest activity of more than 80% at pH 4.5 to 6.5 with the optimal activity at pH 5.0. In addition, exceed 70% of the activity is acquired after 12 h incubation with a pH scope of 3.0–8.0. In particular, the TanF after incubation in the entire pH range (2.0–9.0) for the present study reserves more than 40% of its activity (Figure 5B), manifesting the advisable stability of present tannase under pH conditions.

### Effects of ions on the activity of TanF

**FIGURE 6:**
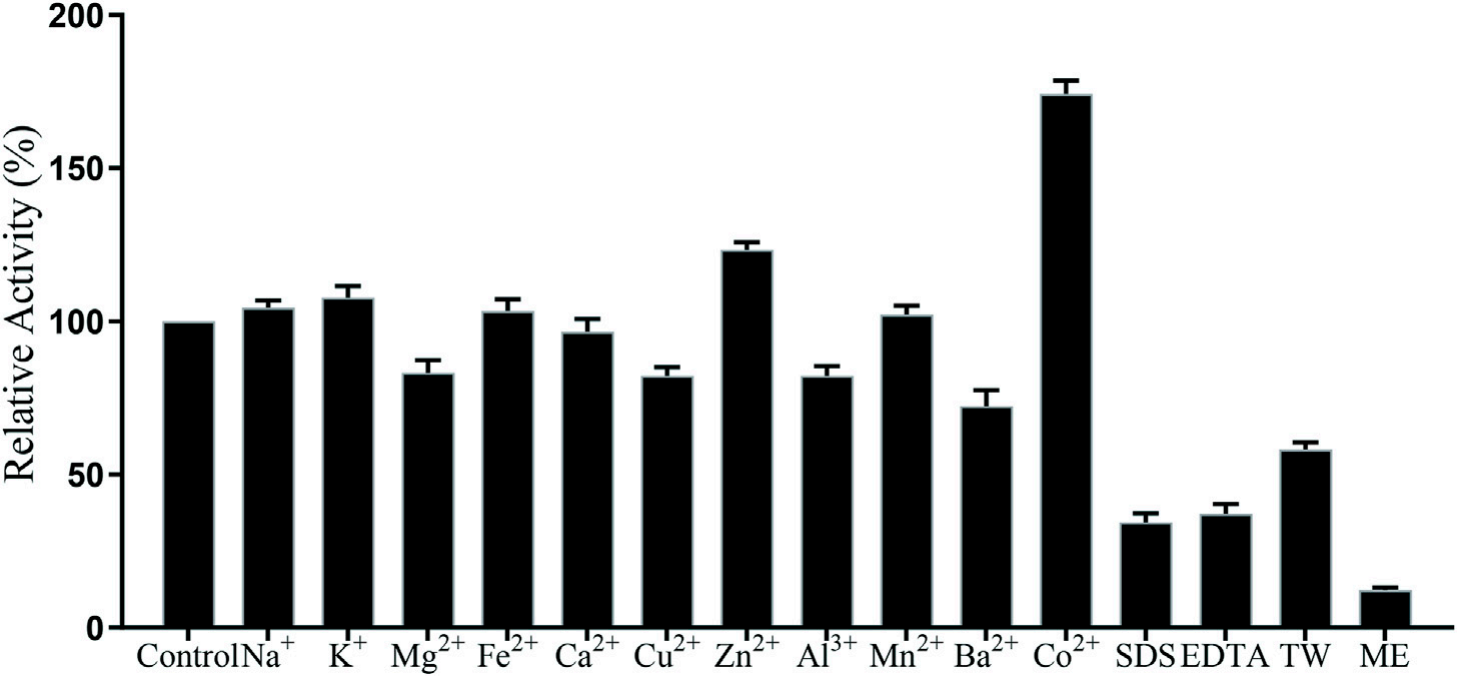
Effects of metal ions and chemicals on the TanF activity. TW: tween 80; ME: 2-hydroxy-1-ethanethiol. Data are shown as mean ± standard deviation, *n* = 3.

In this paper, the effect of metal ions on TanF activity has been investigated, with the results shown in Figure 6. As demonstrated in Figure, except for Ba^2+^, 1 mM other metal ions have no strong inhibitory effect on TanF activity. The activities of TanF in the existence of Co^2+^ is 180.6% of the control group. However, with 1 mM SDS and EDTA, the relative activity of the enzyme is significantly reduced to less than 30%. In addition, in the presence of 1 mM ME (2-hydroxy-1-ethanethiol), the activity of TanF is strongly inhibited, and the relative activity is significantly reduced to 15.6%. It is reported that ME exhibited significant activity inhibition due to its ability to effectively break the disulfide bond in tannase structure. Therefore, TanF shows tolerance to most high metal ion, and Co^2+^ can be acted as a favorable activator of TanF.

### Substrate specificity of TanF

**FIGURE 7:**
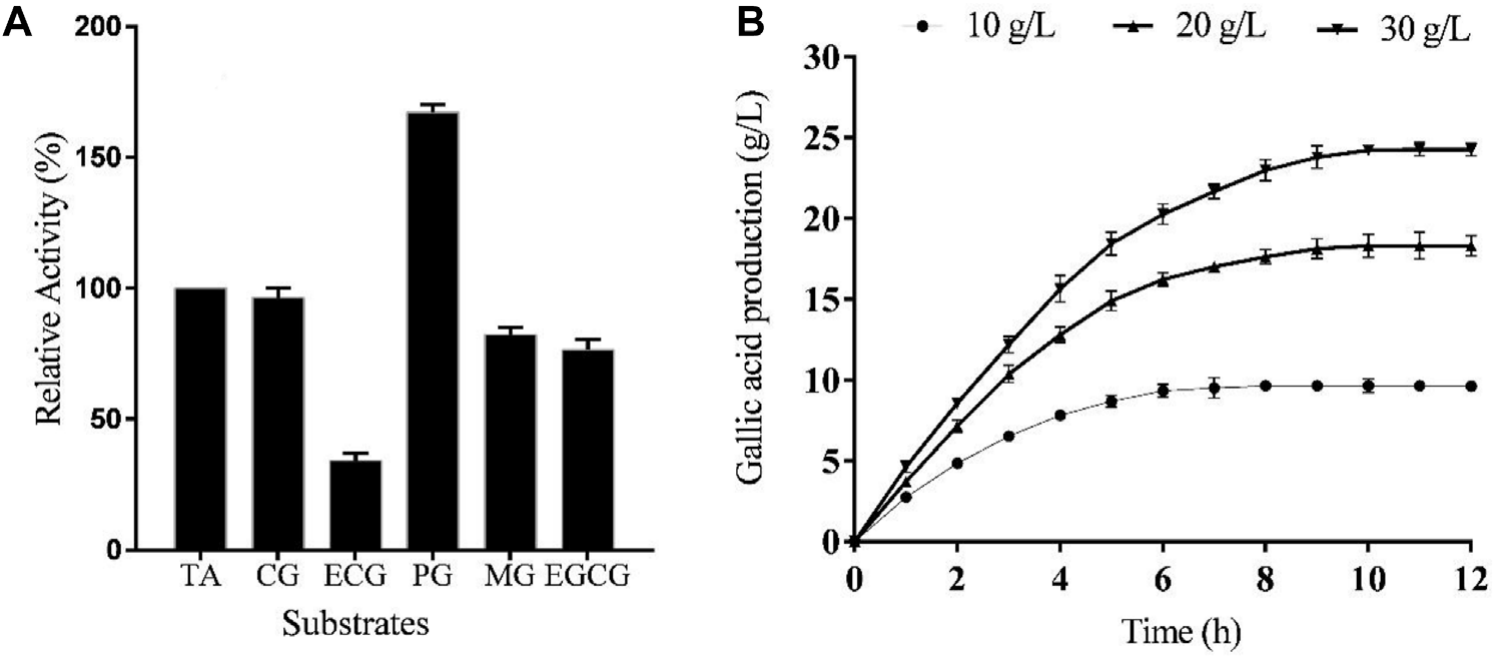
**(A)** TanF activities based on various substrates (methyl gallate (MG), epicatechin gallate (ECG), propyl gallate (PG), catechin gallate (CG), epigallocatechin gallate (EGCG), and tannic acid (TA)).**(B)** Gallic acid changes from tannic acids with different concentrations, *n* = 3.

In order to investigate TanF’s substrate specificity, the purified tannase was subjected to incubation with various gallate esters. The results of this study are presented in Figure 7A. It is evident from the table that TanF exhibits a specific ability to decompose a diverse range of substrates, which highlights its multifunctional activity. Notably, TanF demonstrated superior activity on synthetic substrate PG, while its activity on some natural substrates, including CG, MG, and EGCG, was considerable.

### Gallic acid production by TanF from tannic acid

As shown in Figure 7B, the final gallic acid contents from TA solutions with different concentrations were 9.65 g/L, 18.32 g/L, and 24.23 g/L, respectively. The gallic acid yields from 10 g/L TA and 20 g/L TA were 0.97 g/g and 0.92 g/g, respectively, while the yield from 30 g/L TA as the substrate decreased to 0.81 g/g. Thus, the appropriate TA concentration for gallic acid production was 20 g/L. A final gallic acid content of 18.32 g/L was obtained after 10 h reaction catalyzed by TanF.

## Discussion

Traditionally, industrial tannases were mostly derived from fungi. Recently, many bacterial tannases have also been well characterized (Ebune et al., 1995; Ebune et al., 1995; Tripathi and Mishra, 2007; Lomascolo et al., 2012). However, tannases from yeast strains were still rare. As shown in Table 1, tannase activity of the yeast strain Y71 was higher than those of *A. niger*, *Cyberlindnera rhodanensis*, but lower than those of *Sporidiobolus ruineniae* and *Rhodosporidium diobovatum.* Notably, the tannase of strain Y71 was extracellular, which facilitates the purification and application. Meanwhile, potential resources in the form of uncharacterized tannases from yeast need to be developed. By comparing the sequences of TanF and 11 representative tannases, it can be seen directly that the current enzyme sequence contains a conserved domain, namely Gly-X-Ser-X-Gly (shown in the red box of Figure 1), which is a typical as well as common amino acids of serine hydrolase, showing the highest conservation among all tannase sequences of bacteria and fungi (Zhang et al., 2019).

**TABLE 1:** Tannase activities from fermentation of wild yeasts.

Microorganisms	Activities (U/mL)	Locations	Ref
*Emericella nidulans*	<0.5	extracellular	Gonalves et al. (2011)
*Aspergillus niger*	1.55	extracellular	Shao et al. (2020)
*Sporidiobolus ruineniae*	29.8	cell-associated	Kanpiengjai et al. (2020)
*Kluyveromyces marxianus*	-	extracellular	Mahmoud et al. (2018)
*Cyberlindnera rhodanensis*	11	cell-associated	Iwamoto et al. (2008)
*Rhodosporidium diobovatum*	26.4	extracellular	Pan et al. (2020)
*Debaryomyces hansenii*	14.3	extracellular	This study

Most tannase hydrolysis processes occur at increased temperatures, which is seen as an advantage for soybean meal biodegradation (Sabu et al., 2005). However, some yeast, bacteria (Jana et al., 2014), and mold tannases (Sabu et al., 2005; Sivashanmugam and Jayaraman, 2011) such as *Aspergillus flavus*, *Penicillium chrysogenum*, *A. niger*, and *A. oryzae* (Inoue et al., 2016; Wang et al., 2020) have been shown to display the highest activity at about 40°C. Tannase TanF exhibits favorable stability under temperatures below 40°C, maintaining 78.6% activity even after 2 h of incubation at 40°C.

It is reported that the optimal pH of most tannases from bacteria is approximately 7.0–9.0, while most fungal tannases possess superior activity at pH 6.0. For example, TanF from *Kluyveromyces marxianus* exhibits an optimal pH of 4.5, as shown in Table 2, and displays favorable stability at pH 4.0–4.5. In comparison, tannases from other sources such as *Aspergillus spinosus* (pH 5.0–6.0) and *E. nidulans* (pH 4.0–5.0) show limited pH stability (El-Tanash et al., 2011; Gonalves et al., 2011). Tannase from *Candida* sp. has an optimizing pH of 6.0 (Raghuwanshi et al., 2011; Mahmoud et al., 2018) and maintains high catalytic activities in a narrow pH range of approximately 5.0–7.0 (Ragan, 1986; Schofield et al., 2001; Fuller and Tomé, 2005; Canbolat et al., 2007; Buzzini et al., 2008; Petitot et al., 2009). Efficient and stable tannases at low pH are rare, despite comparisons of tannases from bacteria and fungi (Ebune et al., 1995; Tripathi and Mishra, 2007; Lomascolo et al., 2012; Mejicanos et al., 2016). Yeast tannases have been studied in only a few species, including *S. ruineniae*, *Candida* sp., *Aureobasidium*, *B. adeninivorans*, and *K. marxianus* (Lacassagne et al., 1988; Nair and Duvnjak, 1990; Salem et al., 2005; Hao et al., 2020; Xue et al., 2020). Because both the product and substrate of tannase are acidic, TanF tannase characterized in the present paper can be applied as a potential tool for soybean meal biodegradation due to its satisfying pH stability.

**TABLE 2:** Biochem87ical properties of TanF and reported tannase.

Microorganisms	Optimal pH/Temperature (°C)	pH-stable range	Ref
*Emericella nidulans*	5.0/45	4.0–5.0	Gonalves et al. (2011)
*Aspergillus phoenicis*	6.0/60	2.5–7.0	Gonalves et al. (2011)
*Aspergillus niger*	6.0/80	3.0–8.0	Shao et al. (2020)
*Rhodotorula glutinis*	6.0/40	5.0–8.0	Ong et al. (2022)
*Sporidiobolus ruineniae*	7.0/40	5.0–9.0	Kanpiengjai et al. (2020)
*Kluyveromyces marxianus*	4.5, 8.5/35	4.0–6.0	Mahmoud et al. (2018)
*Lactobacillus plantarum*	8.0/40	7.0–9.0	Iwamoto et al. (2008)
*Debaryomyces hansenii*	5.0/50	3.5–7.0	This study

With the alternative protein source lacking, some unconventional non-food protein resources have gain great attention. Rapeseed dregs, the solid residue extracted from rapeseed oil, was abundant in vitamins and well-balanced amino acids (Ebune, et al., 1995; Jeong et al., 2013; Pustjens et al., 2013). However, the application of rapeseed protein in animal feeds was severely restricted owing to the existence of tannins, which accounted for 0.6–3% of the dry weight. Tannins affecting the absorption of nutrients and possibly causing toxic effects to poultry, becoming the main anti-nutritional factor in rapeseed meal. Therefore, the removal of tannins using strain processing was one of the focus issues. TanF obtained from mangroves with salt tolerance can be another choice to process the unconventional resources in marine aquatic feed.

TanF demonstrated considerable activity on natural substrates, including CG, MG, and EGCG. The substrate specificity makes TanF suitable for tea leaves treatment. In previous studies, green tea leaves treated with tannase resulted in a higher concentration of theaflavin compared to pure withered leaf fermentation, indicating its potential for improving the tea’s quality. Tannase treatment also leads to the hydrolysis of EGC, GA, ECG, and EGCG, resulting in a significant reduction in tea cream formation and an increase in antioxidant activity (Liang et al., 2022). Furthermore, tannase treatment improves the sensory characteristics of black tea infusions, including the intensity of color and the absence of cream formation. In Hibiscus tea, tannase treatment decreased esterified catechins by 8.91% while increasing non-esterified catechins by 19.76%. Total phenolic compounds in Hibiscus tea also increased significantly by 8.6% due to tannase treatment (Sayed, 2023). Therefore, tannase represents a potential means of producing low-astringency Hibiscus tea while also enhancing its quality. Tea leaves treatment can be new direction of application of TanF.

Gallic acid synthesis using tannase producing microorganisms has been studied extensively. *Candida* sp. and *K. marxianus* are two yeast species that have shown potential for the production of extracellular tannase (Bell, 1984; Lorusso et al., 1996; Petitot, et al., 2009). It is important to note that *Aspergillus* sp. has been widely used for tannase production in previous studies, but their ability to convert tannic acid to gallic acid has not been thoroughly evaluated. Additionally, it has been suggested that the accumulation of gallic acid is not directly associated with the tannase yield of the organism, indicating that *Aspergillus* sp. may only serve as a tannase producer due to its endogenous metabolism. (Salem et al., 2005; Canbolat et al., 2007). Similar findings were observed in studies involving *B. thurangiences* and *A. Pullulans* (Abebe et al., 2007; Xue et al., 2020). The gallic acid yields of these microorganisms were too low to perform commercial production.

Cell wall-associated tannases were better choice for gallic acid synthesis. *A. niger* Aa-20 and *A. aculeatus* DBF9 have been reported to produce 7.64 g/L and 6 g/L gallic acid, respectively, from tannic acid (Nair and Duvnjak, 1990; Salem et al., 2005). In a recent study, *B. subtilis* AM1 and *Lactobacillus plantarum* CIR1 produced the maximum levels of gallic acid in non-optimized conditions during anaerobic fermentation of 10 g/L tannic acid, with final contents of 2.41 and 2.37 g/L, respectively (Hao et al., 2020). Another cell wall-associated tannase from yeast *S. ruineniae* can appears to be more efficient for gallic acid production, with a final content of 11.8 g/L (Kanpiengjai et al., 2020).

In this study, concentrated recombinant TanF was used as the catalyst for gallic acid synthesis. The concentration of substrate can be up to 20 g/L. Meanwhile, the yield was improved to above 0.90 g/g, due to the absence of endogenous metabolism in the live cell. Actually, the final gallic acid production achieved 18.32 g/L, much higher than those in studies above mentioned. Therefore, TanF has been demonstrated to be an efficient tool for the preparation of gallic acid. Enzyme modification and process optimization will be carried out to prompt the application of TanF in commercial scale.

## Data Availability

The original contributions presented in the study are included in the article/supplementary material, further inquiries can be directed to the corresponding authors.
